# Removal efficiencies of seven frequently detected antibiotics and related physiological responses in three microalgae species

**DOI:** 10.1007/s11356-024-32026-5

**Published:** 2024-01-26

**Authors:** Gabriele Frascaroli, Joanne Roberts, Colin Hunter, Ania Escudero

**Affiliations:** 1 Department of Civil Engineering and Environmental Management, School of Computing, Engineering and Built Environment, Glasgow Caledonian University, Cowcaddens Road, Glasgow, G4 0BA UK; 2 Department of Applied Science, School of Computing, Engineering and Built Environment, Glasgow Caledonian University, Cowcaddens Road, Glasgow, G4 0BA UK

**Keywords:** Microalgae, Phycoremediation, Emerging contaminants, Biosorption, Active removal, Pigment overproduction

## Abstract

The main objective of this study is to investigate the effect of mixtures of seven widely used human antibiotics (ciprofloxacin, clarithromycin, erythromycin, metronidazole, ofloxacin, sulfamethoxazole, and trimethoprim) on the growth, pH, pigment production, and antibiotics removal of three microalgal species (*Auxenochlorella protothecoides*, *Tetradesmus obliquus*, and *Chlamydomonas acidophila*). Batch assays were conducted with media with antibiotic mixtures at 10, 50, and 100 μg L^−1^ for each antibiotic. The three microalgae species effectively removed the antibiotics without any growth inhibition, even when exposed to the highest antibiotic concentrations. Biosorption was reported as the primary mechanism for ciprofloxacin, clarithromycin, metronidazole, and ofloxacin, with up to 70% removal, especially in *A. protothecoides* and *C. acidophila*. *A. protothecoides*, a species never investigated for antibiotic removal, was the only microalgae exhibiting bioaccumulation and biodegradation of specific antibiotics, including sulfamethoxazole. Furthermore, in media with the highest antibiotic concentration, all three species exhibited increased chlorophyll (up to 37%) and carotenoid (up to 32%) production, accompanied by a pH decrease of 3 units. Generally, in the present study, it has been observed that physiological responses and the removal of antibiotics by microalgae are interlinked and contingent on the antibiotic levels and types.

**Supplementary Information:**

The online version contains supplementary material available at 10.1007/s11356-024-32026-5.

## Introduction

Since the early twentieth century, antibiotics have been extensively and successfully used to treat infectious diseases, revolutionising modern medicine. However, the increasing use and misuse of antibiotics have led to their constant release into the environment, with negative impacts on ecological and human health (Kümmerer 2003). When administrated, antibiotics are not completely metabolised, and via excreta, residual fractions and their metabolites reach local wastewater treatment plants (WWTPs) (Kümmerer and Henninger 2003), where they may remain active (Roberts and Thomas 2006). High concentrations of antibiotics, not removed by standard treatment approaches, have been identified in several studies of effluent wastewater (WW) released directly in aquatic environments (Frascaroli et al. 2021). After discharge into rivers, lakes, and other aquatic environments, antibiotics may disturb the ecological balance of habitats and drive the spread of antimicrobial resistance (AMR) (Roberts 2002). In a recent systematic review, we proposed a list of the seven most concerning antibiotics commonly found in influents and effluents of WWTPs (Frascaroli et al. 2021). According to the list, ciprofloxacin, clarithromycin, erythromycin, metronidazole, ofloxacin, sulfamethoxazole, and trimethoprim pose significant risks due to their widespread presence, concerning concentrations, high RQ values, and resistance to removal treatments. Of particular concern are ciprofloxacin, clarithromycin, and ofloxacin, frequently detected above predicted no-effect concentrations for resistance selection. On these grounds, further action is required to study alternatives to standard WWTPs that could better remove these antibiotics in WW.

Recently, there has been increased interest in biological methods for the removal of organic and inorganic micropollutants utilising microalgae (Mondal et al. 2019). As microalgae are ubiquitous in freshwater and marine environments, these microorganisms have developed extensive abilities to tolerate severe fluctuations in pH, temperature, and organic and inorganic pollutants (Lau et al. 1999; Lei et al. 2002). For these reasons, microalgae-based technology has been widely studied to treat municipal (Cho et al. 2011; Foladori et al. 2018) and industrial effluents (Chinnasamy et al. 2010; Van Den Hende et al. 2014). Municipal WW has been used successfully to grow species such as *Auxenochlorella protothecoides*, showing remarkable treatment abilities (Zhou et al. 2012). Moreover, lately, the abilities of microalgae have become a source of interest in testing antibiotics removal in WW (Kiki et al. 2020; Michelon et al. 2022). For example, the species *Tetradesmus obliquus*, previously *Scenedesmus obliquus*, has proved highly effective in the degradation of clarithromycin (100.00 ± 1.10%), ciprofloxacin (78.16 ± 2.56%), and ofloxacin (44.78 ± 10.72%) from raw WW (Zhou et al. 2014). Similarly, *Chlamydomonas acidophila* exhibited high removal efficiencies of the antibiotics erythromycin and clarithromycin by 65–93% and 50–64%, respectively (Escudero et al. 2020). Microalgae can remove antibiotics through biosorption, involving passive binding of the molecules on their surface, bioaccumulation, actively taking antibiotics into the cells, and biodegradation through enzymatic processes (Mustafa et al. 2021). Currently, there is still a limited comprehension of the removal pathways that occur when algae interact with antibiotics. Specifically, there is a need for a more evident differentiation between passive biosorption and active removal, which includes bioaccumulation and degradation.

Although the resistance capacity of microalgae toward aquatic pollutants is well documented (Palmer 1969; Rai et al. 1981; Wu et al. 2022), the stress generated by antibiotics may influence their removal behaviour and WW treatment performance. Moderate stress levels induced by antibiotics can stimulate the production of cellular compounds like chlorophylls and carotenoids. In contrast, severe oxidative stress can hinder growth, decrease photosynthesis, and cause cell death (Xiong et al. 2017; Mao et al. 2021). Some studies have shown the toxic effects of antibiotics on species such as *Microcystis aeruginosa* (Qian et al. 2012) or *Chlorella vulgaris* (Dong et al. 2020). For instance, Xiong et al. (2020a) scrutinised the alterations in the structure and composition of lipids and proteins of *Pseudokirchneriella subcapitata* exposed to various concentrations of levofloxacin and sulfamethoxazole. To date, much research focuses on the toxicity effect of single antibiotics on a few model microalgae species, typically never used for treatment purposes. Therefore, further research is required to understand the response mechanisms triggered by the presence of mixtures of antibiotics in microalgae proven effective in treating WW. Furthermore, there is still a poor understanding of the dynamics occurring during the interaction between antibiotics and algae and information gaps regarding how the responses to stress may influence antibiotic removal.

The main objective of this study is to investigate the algal physiological and metabolic responses toward stress and how those responses may influence the removal of antibiotics. For that purpose, three microalgae used for WW treatment were selected (*A. protothecoides*, *T. obliquus*, and *C. acidophila*) to evaluate the effect of mixtures of seven widely used human antibiotics (ciprofloxacin, clarithromycin, erythromycin, metronidazole, ofloxacin, sulfamethoxazole, and trimethoprim) on the growth, pH, pigment production, and antibiotics removal.

## Materials and methods

### Microalgae strains and culture growing conditions

The strains of *Auxenochlorella protothecoides* (CCAP 211/7A), *Tetradesmus obliquus* (CCAP 276/6B), and *Chlamydomonas acidophila* (CCAP 11/133) were purchased from the Culture Collection of Algae and Protozoa (Oban, UK). The cultivation was performed with sterile BG-11 medium in 1L bottles equipped with a filtered bubbling system to allow the continuous suspension of the cells. The bottles were illuminated by a fluorescent lamp, at a light intensity of 4000 lux, with a 16 h/8 h light/dark interval, and the temperature was maintained at 21 ± 3 °C.

### Chemicals and reagents

Ciprofloxacin (CIP), clarithromycin (CLA), ofloxacin (OFL), and trimethoprim (TMP) were purchased from Sigma-Aldrich (St. Louis, USA). Erythromycin (ERY) and metronidazole (MDZ) were obtained from Acros Organics (Geel, Belgium). Sulfamethoxazole (SMX) was purchased from MP Biomedics (Santa Ana, USA). The purity of the antibiotics was above 98%, except for clarithromycin (>95%). Antibiotic solutions to spike in the tests were prepared freshly at the beginning of the experiment in ultrapure water and formic acid (1%).

HPLC grade methanol, acetonitrile, and formic acid were obtained from Fisher Scientific (Waltham, USA). HEPES and methanol (purity > 99.5%) used for the analysis of the pigments were purchased from Sigma-Aldrich (St. Louis, USA). Solutions of these reagents were stored in optimal conditions for not more than 2 weeks. All solutions and the media were prepared using water produced by an ultrapure water system (Elga Purelab, High Wycombe, UK).

### Experimental design

The effects of a mixture of antibiotics at different concentrations and their removal by microalgae were studied in batch culture experiments. The microalgae cells were harvested and separated by centrifugation from the mother culture when they reached the exponential growth phase and inoculated in 500 mL Erlenmeyer flasks to achieve an initial concentration of 2.5 × 10^6^ cells mL^−1^. The flasks were filled with 300 mL of sterilised modified BG-11 medium at pH 7, in which the concentration of the three predominant nutrients was adjusted as follows: 60 mg L^−1^ of NH_4_^+^-N, 1.5 mg L^−1^ of NO_3_−N, and 5 mg L^−1^ of PO_4_^3−^−P, to reach values typically found in WW (Supplementary information, Table S1). All samples were incubated in triplicate in a shaking incubator Incu-Shake FL24-1 (SciQuip, Newtown, UK) maintained at 120 rpm, 23 ± 3 °C, under an average natural white LED illumination intensity of 4000 lux, with a 16 h/8 h light/dark cycle.

The batch experiment aimed to simulate an aquatic environment where a mixture of antibiotics may be present at high concentrations, such as in a municipal sewage system. In WW, the highest concentrations of the seven antibiotics selected for the experiment can range from 1 to 90 μg L^−1^ (Frascaroli et al. 2021). Therefore, to replicate three high-stress scenarios in this experiment, we selected three different concentrations of antibiotics: 10, 50, and 100 μg L^−1^, which represent the initial concentrations per antibiotic. Solutions of the seven different antibiotics were prepared at 1 mg mL^−1^ in ultrapure water with 1% formic acid. These were combined to create the three mixtures at concentrations of 10, 50, and 100 μg L^−1^ per antibiotic. Flasks with no antibiotics spiked were used as a control. Three replicas for each treatment were arranged.

In order to assess the influence of abiotic factors such as hydrolysis and photolysis, an experiment was conducted employing a solution containing a mixture of the seven antibiotics, each at concentrations of 50 μg L^−1^. These experiments were carried out using a sterilised medium devoid of algae. Hydrolysis was measured in flasks exposed to the same light condition as the flasks with algae, while photolysis was assessed in flasks covered with a black nitrile film. Three replicas were prepared for each treatment, also in this case.

The number of cells was calculated on days 0, 1, 2, 3, 6, and 9 of incubation; the concentration of chlorophylls and carotenoids at day 9. The pH values and antibiotics concentrations on the media were analysed on days 0, 3, 6, and 9. The experimental setup is summarised in Table 1.

**Table 1:** Experimental setup: in the biotic experiment, microalgae were exposed to three different mixtures of antibiotics (Mix 10, Mix 50, and Mix 100) and compared with controls. In the abiotic experiment, hydrolysis and photolysis of antibiotics were calculated at 50 μg L^−1^ of each antibiotic

Condition	Name	Each antibiotic concentration (μg L^−1^)	Total 7 antibiotic concentration (μg L^−1^)	Inoculum of the alga	Light condition
Biotic	Control	0	0	Yes	Light
	Mix 10	10	70	Yes	Light
	Mix 50	50	350	Yes	Light
	Mix 100	100	700	Yes	Light
Abiotic	Hydrolysis	50	350	No	Dark
	Photolysis	50	350	No	Light

### Analytical procedures

#### Determination of antibiotics concentration

At days 0, 3, 6, and 9 of incubation, 2 mL from each sample was collected and centrifuged at 13,000 rpm for 15 min. The supernatant was filtered through a 0.2 μm PVDF syringe filter (Whatman™, Maidstone, UK). The samples were further treated by solid-phase extraction (SPE) using Oasis HLB 3 cc cartridges (60 mg sorbent, 30 μm) (Waters™ Milford, USA). Samples were reconstituted in acetonitrile/water (10:90) and stored for no more than 2 weeks at −20 °C until analysis. SPE method, recoveries, matrix effect, and limits of detection for each antibiotic are described in Supplementary information (Fig. S1, Table S2–4).

The antibiotic concentrations in the media were analysed using liquid chromatography coupled with mass spectrometry (LC-MS) using a Thermo Scientific Q-Exactive Orbitrap mass spectrometer equipped with an Accucore™ C18 HPLC Column (150 × 2.1 mm) (Thermo Fisher Scientific, USA). The mass spectrometer was fitted with a Dionex Ultimate 3000 RS pump, Dionex Ultimate 3000 RS autosampler (temperature controlled at 10 °C), and Dionex Ultimate 3000 RS column compartment (temperature controlled at 30 °C). The operating software was Chromeleon, Xcalibur, and Tracefinder. The antibiotics were detected in positive mode using the transitions described along with the mass spectrometry characteristics in Supplementary information (Table S5).

Abiotic removal, consisting of photolysis and hydrolysis, was assessed by measuring the decline in antibiotic concentrations over time using sterilised media devoid of algae. On the other hand, the presence of microalgae might introduce three distinct mechanisms for antibiotic removal: biosorption, bioaccumulation, and biodegradation. The antibiotic removal due to biosorption into the microalgae biomass was calculated as the difference between the abiotic and the biotic concentration in the media. A maximum contact time of 10 min was set between the addition of the antibiotics and the media collection for the quantification. On the other hand, bioaccumulation and biodegradation—whose distinction was not the object of the study—were calculated by measuring the decrease in antibiotic concentration during the incubation time and collectively considered as active removal.

#### Determination of microalgae growth and the pH of the media

Microalgae growth was monitored throughout the experiment to assess the health of cultures and identify any inhibition caused by the mixtures of antibiotics. The number of cells was counted through Celeromics Technologies SL Micro Counter® (Valencia, Spain) at ×20 under an optical microscope (Brunel Microscopes Ltd, Chippenham, UK).

The variation in the pH of the media was measured through a pH meter (Accument® Basic AB 15, Fisherbrand, UK).

#### Determination of chlorophylls and carotenoids

Chlorophylls and carotenoids are critical indicators for assessing the physiological state of cells since they act as a protective agent during a stress event (da Silva Rodrigues et al. 2020). The pigment content was measured following the protocol of Xiong et al. (2017), with some modifications. Briefly, 2 mL samples were gathered and centrifuged at 13,000 rpm for 15 min. The supernatant was collected and maintained for the antibiotic analysis, and the pellet was suspended in 2 mL of HEPES (50 mM, pH 7.0) and centrifuged again at the same speed. The supernatant was discarded, and the pellet was re-suspended in 2 mL of 90% methanol and incubated at 60 °C for 30 min in an ultrasonic bath. After centrifugation at 13,000 rpm for 5 min, the absorbance of the supernatant was measured using a Genesis 10S UV-Vis spectrophotometer (Thermo Scientific, Waltham, USA) at 665, 652, and 470 nm. The concentration of chlorophylls and carotenoids was calculated following Eqs. (1, 2, 3):

1 $$\textrm{Chlorophyll}\ \textrm{a}\ \left(\textrm{ChlA},\upmu \textrm{g}\ {\textrm{L}}^{-1}\right)=\left(16.82\ {\textrm{OD}}_{665}-9.28\ \textrm{OD}_{652}\right)\times 1000$$

2 $$\textrm{Chlorophyll}\ \textrm{b}\ \left(\textrm{ChlB},\upmu \textrm{g}\ {\textrm{L}}^{-1}\right)=\left(36.92\ {\textrm{OD}}_{652}-16.54\ {\textrm{OD}}_{665}\right)\times 1000$$

3 $$\textrm{Carotenoids}\ \left(\upmu \textrm{g}\ \textrm{L}^{-1}\right)=\left(\frac{\left(1000\ {\textrm{OD}}_{470}\right)-\left(1.91\ \textrm{ChlA}\right)-\left(95.15\ \textrm{ChlB}\right)}{225}\right)\times 1000$$

### Data analysis

Statistical analyses were performed using Microsoft Excel 2016 (Microsoft Corporation, WA, USA) and SPSS Statistics V26.0 (IBM SPSS Statistics, Armonk, USA). Independent *t*-test, based on Levene’s test for equality of variances, and one-way ANOVA, following the Bonferroni correction method, were used to determine significant differences at 95 and 99% confidence levels (*p* < 0.05 and 0.01). Standard deviation values were calculated and referred to at least three data points.

## Results and discussion

### pH variation and microalgae growth

The pH variation in response to antibiotics was monitored in the media with and without algae during the incubation. No significant variation (*p* > 0.05) was observed in the experiment in abiotic conditions, without algae and with antibiotics.

**Fig. 1:**
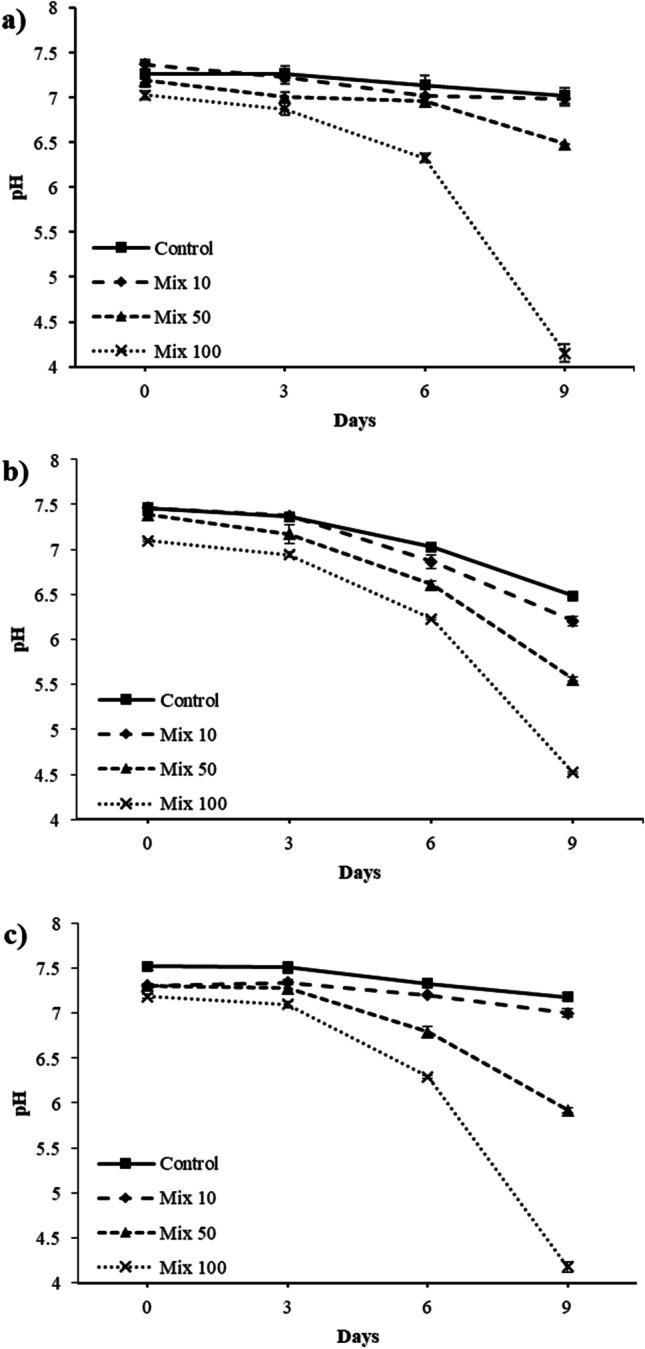
Over time pH variation in the media of *A. protothecoides* (**a**)*,* *T. obliquus* (**b**) *and* *C.* *acidophila* (**c**) exposed to 4 mixtures of antibiotics, 0 μg L^*−*1^ (control), 10 μg L^−1^ (Mix 10), 50 μg L^−1^ (Mix 50), and 100 μg L^*−*1^ (Mix 100)*. *Figures show the average data of two replicas

Figure 1 shows a decrease in the pH over time occurring in all the media in which the microalgae were spiked with antibiotics. This pattern seems to be linked and proportional to the concentration of antibiotics in the media for the three algae species studied. In the media with the highest antibiotics concentration (100 μg L^−1^), after 9 days of cultivation, the pH values dropped by 2.6, 2.8, and 3 for *T. obliquus*, *A. protothecoides*, and *C. acidophila*, respectively (Fig. 1b, a, c). In contrast, no significant variation (*p* > 0.05) was observed in the abiotic media without algae or controls without antibiotics except for the control sample of *T. obliquus* (Fig. 1b), where the pH value decreased slightly during the incubation period.

Although the buffer capacities of some microalgae have been previously reported (Wildman et al. 1974), there is a lack of literature on the potential causes of pH decrease. Based on the results obtained in the current study, it seems that the variation in the pH is not aimed at the formation of a more suitable environment for the microalgae, but it is due to a switch in the algal metabolism triggered by antibiotics. More specifically, it seems that the presence of organic compounds in the media might activate and increase the heterotrophic activity in the system (Markou et al. 2021), which could lead to a higher production of CO_2_ and a simultaneous decrease in gas consumption. This CO_2_ produced would dissolve in the water in the form of carbonic acid, lowering the pH of the media. Algae are recognised to overlap the various nutritional modes, switching between phototrophic and heterotrophic metabolisms depending on environmental conditions (Kaplan et al. 1986). In the current study, the pH variation was a presumed response of the three microalgae to the presence of antibiotics. This response appeared directly proportional to the levels of the exogenous compounds in the media: the higher the concentration of antibiotics, the higher the drop in the pH. Therefore, it seems that the presence of organic antibiotics drove a shift to a heterotrophic state of the system, consequently causing a decrease in the pH of the media.

Regarding the microalgae growth, the change in the pH caused by antibiotics did not influence *A. protothecoides* and *T. obliquus*. No significant difference (*p* > 0.05) was observed in the cell concentration for these two species regardless of the antibiotic concentrations in the media (Supplementary information, Fig. S2). However, the growth rates of *A. protothecoides* in Mix 50 and Mix 100 reached values of 0.05 d^−1^ compared to the 0.04 d^−1^ of the control, although not statistically significant (*p* > 0.05). The presence of antibiotics and their hypothetical use as a carbon source may explain the slightly higher value for this species, which has proven to grow better in mixotrophic conditions (Markou et al. 2021). The same was observed for *T. obliquus*, with growth rates in the media with antibiotics of 0.10 d^−1^, compared to the control (0.08 d^−1^), although no statistically significant difference (*p* > 0.05) was reported.

**Fig. 2:**
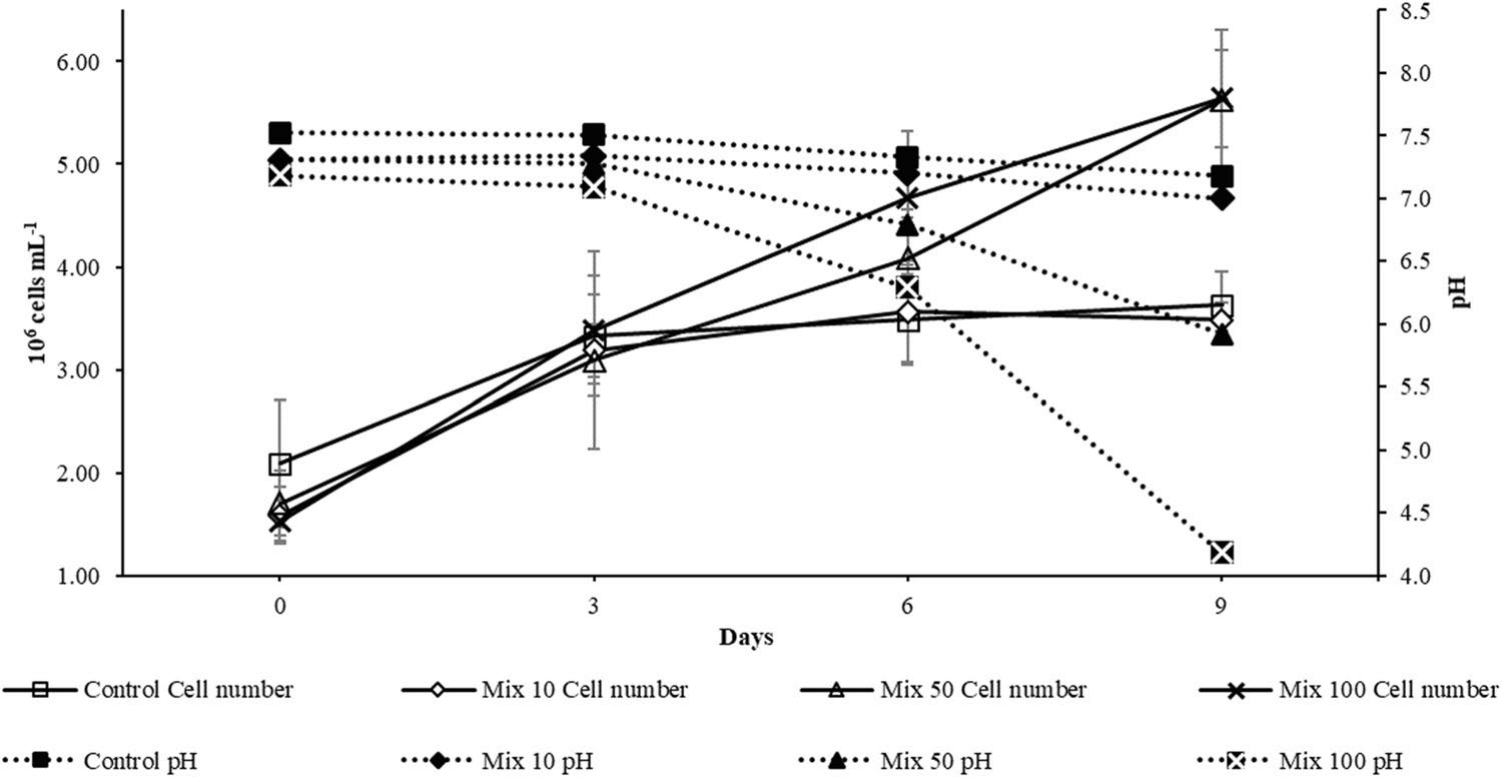
Growth curves of *C. acidophila* exposed to the mixtures of antibiotics 0, 10, 50, and 100 μg L^−1^ plotted with curves representing the change in the pH in the same media. The figure shows the average data of three samples and at least one measurement

On the other hand, a significant increase (*p* < 0.01) in the cell number of *C. acidophila* was reported in the media with the highest concentrations of antibiotics (Mix 50 and Mix 100), as shown in Fig. 2.

*C. acidophila* is an acidophilic species isolated from acidic strip-mine ponds with pH values around 2–3 (Negoro 1944). Therefore, the increased growth rate in Mix 50 and Mix 100 (0.13 and 0.14 d^−1^, respectively) might be related to the increased acidity of these media.

The presented results, therefore, show that the three algae were not negatively affected by the antibiotics and proved to endure levels typically found in WW. Other authors have also confirmed that microalgae of the same genera examined in this experiment tolerate high concentrations of antibiotics. A previous study showed that the green algae *Chlamydomonas sp.* Tai-03 coped with concentrations of sulfamethoxazole up to 10 mg L^−1^ with no inhibition (Xie et al. 2019). Similarly, Gojkovic et al. (2019) demonstrated that the growth of the strain RISE of *T. obliquus* was not affected by the presence of 19 pharmaceuticals. By contrast, in Kiki et al. (2020), the growths of *Haematococcus pluvialis*, *Scenedesmus quadricauda*, and *Chlorella vulgaris* increased under 20, 50, or 100 μg L^−1^ of a mixture of ten antibiotics. According to the authors, the reported increase was based on the consumption of pharmaceuticals as a nutritional source of organic compounds.

Although this assumption appears plausible, in the present study, only *C. acidophila* significantly (*p* < 0.01) augmented the cell production in media with higher antibiotics concentration; however, other known factors must be mainly accounted for, such as the pH variation.

### Chlorophylls and carotenoids

**Fig. 3:**
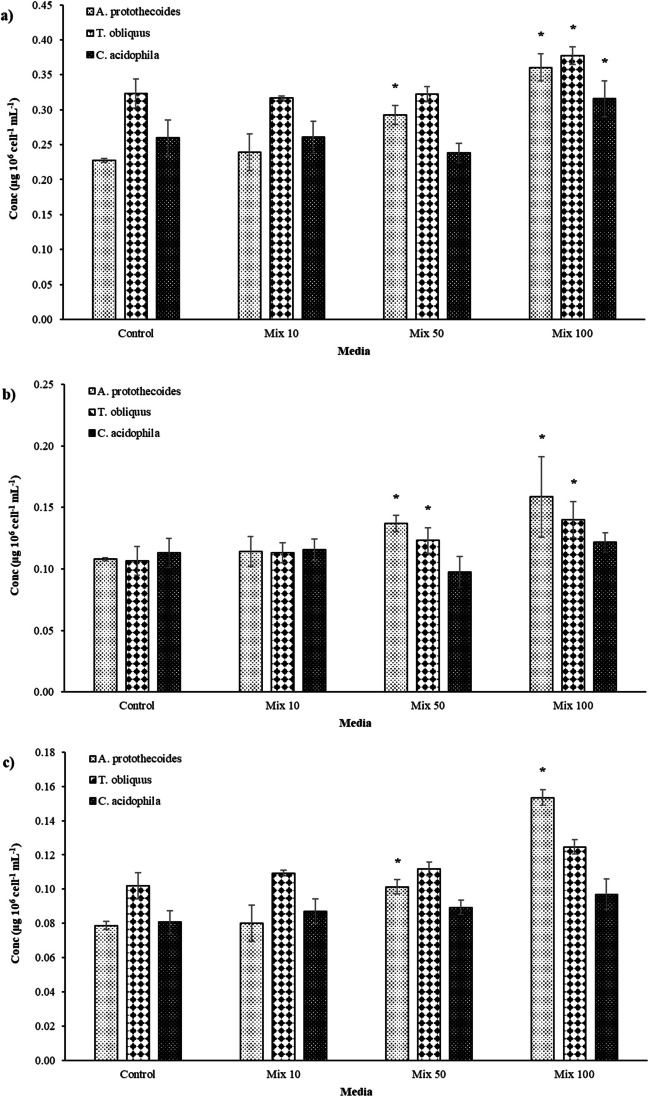
Normalised concentration of chlorophyll A (**a**), chlorophyll B (**b**), and carotenoids (**c**) after 9 days of exposition to three mixtures of antibiotics, compared to the control*.* The figures show the average data of three replicas. * means a statistically significant difference (*p* < 0.05) between the media with the antibiotics (Mix) and the control

Pigments such as chlorophyll A (ChlA), chlorophyll B (ChlB), and carotenoids can be used as indicators of the status of the cells, for the presence of exogenous compounds usually alters the photosynthetic activity of the microalgal cell. Figure 3 shows the effect of mixtures of the 7 antibiotics on the pigment content of the three microalgae studied.

After 9 days of cultivation, differences in the concentration of pigments produced were observed between media with antibiotics at a concentration of 50 and 100 μg L^−1^ and the controls and between different microalgae strains. On the other hand, no significant (*p* > 0.05) overproduction of carotenoids or chlorophylls has been observed in the media with the lowest concentration of antibiotics (Mix 10). In detail, at the end of the experimentation, the three microalgae species showed a significantly higher ChlA content (*p* < 0.05) in Mix 100 compared to the controls, with an increase of 14.4, 17.8 and 36.9%, for *T. obliquus*, *C. acidophila*, and *A. protothecoides* respectively (Fig. 3a). A similar pattern was observed for the same pigment in *A. protothecoides*; at a lower concentration of antibiotics (Mix 50), ChlA increased by 22.3%. Furthermore, *A. protothecoides* was the only species with a higher ChlB content in the presence of 50 and 100 μg L^−1^ of antibiotics (Fig. 3b). These results are consistent with previously published studies. Xiong et al. (2017) reported increased concentrations of chlorophylls in *Chlamydomonas mexicana* and *Micractinium resseri* with the increase in the concentration of enrofloxacin (1, 20, 50, and 100 mg L^−1^). Regarding the carotenoid content, an increase was observed only for *T. obliquus* and *A. protothecoides* when exposed to the highest concentrations of antibiotics*.* The concentration of carotenoids in Mix 50 and Mix 100 was respectively 13.4 and 23.9% higher than the control in *T. obliquus* and 21.2 and 32.1% in *A. protothecoides* (Fig. 3c). Increased content of carotenoids in *T. obliquus* when exposed to sulfamethazine and sulfamethoxazole in a mixture has previously been described (Xiong et al. 2019a).

Chlorophylls and carotenoids play a critical role during stress responses to toxic compounds in the environment, as they act as protective agents in scavenging the reactive oxygen species (ROS) naturally produced in the chloroplasts during stress conditions (Sun et al. 2018). In addition to the complex system for ROS scavenging in chloroplasts, chlorophylls facilitate ROS generation through light-dependent reactions (Edreva 2005). In a previous study, Luo et al. (2015) demonstrated the active process of photo-transformation of benzene via photocatalytic generation of singlet oxygen mediated by active chlorophylls extracted from the algal species *P. subcapitata*.

According to the current results, the overproduction of pigments appeared triggered by the stress induced by antibiotics at concentrations relevant to WWTPs. Overproduced pigments, particularly chlorophylls, may actively degrade the antibiotics or scavenge the ROS caused by them. In general, *A. protothecoides* and *T. obliquus* seemed more sensitive to the presence of antibiotics than *C. acidophila*, producing higher amounts of pigments than the controls when exposed to the antibiotics. Therefore, it seems that *C. acidophila* is more tolerant to the compounds used in this experiment than *A. protothecoides* and *T. obliquus*. It is essential to highlight that pigment overproduction was observed exclusively when the algae were exposed to the highest antibiotic concentrations (Mix 50 and 100), mimicking some of the most severe stress situations in a municipal WWTP. This phenomenon did not manifest at lower antibiotic concentrations (Mix 10), which still hold ecological importance in WW systems. However, it is worth noting that despite the pigment overproduction, no growth inhibition was observed in any of the three species, as previously reported (Fig. 1). Moreover, whether antibiotics might increase the production of pigments in algae, the stress depends on the delicate equilibrium between the scavenging of ROS caused by exogenous compounds and ROS production to degrade them. Additional experiments over a more extended period are required to understand if the constant presence of antibiotics, as in a real WWTP, may further deregulate the production of pigments, causing a long-term inhibition of algae.

### Antibiotics removal

The pathways involved in the removal of antibiotics from WW consist of abiotic hydrolysis and photolysis, and biotic biosorption, bioaccumulation, and biodegradation (Kümmerer 2008). In the present study, the abiotic and biotic removal of the 7 selected antibiotics (CIP, CLA, ERY, MDZ, OFL, SMX, and TMP) was investigated in batch mode in which mixtures of these at WWTPs relevant concentrations were added.

During the incubation, no significant differences (*p* > 0.05) in antibiotic concentrations or constant decreasing patterns were observed in abiotic samples (Table 2), which indicates that these compounds were not removed from the media due to photolysis or hydrolysis. These results are consistent with the literature showing the recalcitrance of most antibiotics used in the present study under abiotic conditions (Loftin et al. 2008; Xiong et al. 2019b).

**Table 2:** Concentrations (μg L^−1^ ± SD) of the antibiotics* in the abiotic experiment on days 0, 3, 6, and 9. The initial concentration spiked for each antibiotic was 50 μg L^−1^. The table shows the average data of three samples and three measurements ± SD

Abiotic dark condition concentrations (μg L^−1^)						
	CIP	CLA	MDZ	OFL	SMX	TMP
Day 0	36.4 (± 1.7)	7.5 (± 3.7)	47.9 (± 1.9)	36.5 (± 5.9)	30.1 (± 3.1)	36.2 (± 2.5)
Day 3	51.9 (± 3.1)	8.4 (± 0.4)	47.6 (± 0.4)	47.3 (± 3.5)	33.4 (± 2.6)	62.7 (± 39.5)
Day 6	37.4 (± 2.2)	5.8 (± 1.0)	46.3 (± 1.1)	43.7 (± 11.7)	31.4 (± 1.3)	53.6 (± 23.7)
Day 9	33.3 (± 1.4)	7.0 (± 2.8)	49.9 (± 0.9)	34.2 (± 2.6)	35.8 (± 4.5)	40.4 (± 5.6)
Abiotic light conditions concentrations (μg L^−1^)						
	CIP	CLA	MDZ	OFL	SMX	TMP
Day 0	34.8 (± 8.5)	7.3 (± 3.0)	44.0 (± 2.6)	36.8 (± 3.6)	37.6 (± 10.4)	33.0 (± 3.6)
Day 3	46.7 (± 2.5)	6.2 (± 0.3)	43.6 (± 1.0)	42.5 (± 3.3)	32.9 (± 2.8)	38.2 (± 7.9)
Day 6	36.1 (± 3.3)	7.4 (± 0.6)	44.6 (± 2.1)	37.5 (± 4.9)	30.4 (± 2.9)	43.0 (± 15.3)
Day 9	31.1 (± 11.3)	8.9 (± 1.8)	47.8 (± 0.7)	29.7 (± 8.0)	28.2 (± 9.8)	38.3 (± 3.4)

*Erythromycin concentrations are not reported as the analytical data was inconsistent, and it became apparent the addition of acid during the SPE degraded the antibiotic (Supplementary Figure S1 and Table S2)

By contrast, all the antibiotics, except for TMP, appeared to be subjected to removal via biosorption, bioaccumulation, or biodegradation at different rates depending on the algae.

The stability of TMP in the abiotic and biotic batches can be explained by the high pK_a_ values of this substance and its low *K*_ow_, which would prevent biomass adsorption, favouring the dissolution of this compound in the aqueous phase (Vassalle et al. 2020).

#### Biosorption

The ability of microalgae to remove antibiotics from WW through biosorption has been demonstrated previously (Chen et al. 2020; Xiong et al. 2020b; Pan et al. 2021). In the present trial, the employed microalgae showed that some antibiotics commonly found in WW could be effectively removed at the first contact by biosorption. Biosorption is the passive process for which the complex variety of functional groups on the surface of the cell wall is immediately engaged as a sorbent to bond exogenous compounds such as antibiotics (Spain et al. 2021).

**Fig. 4:**
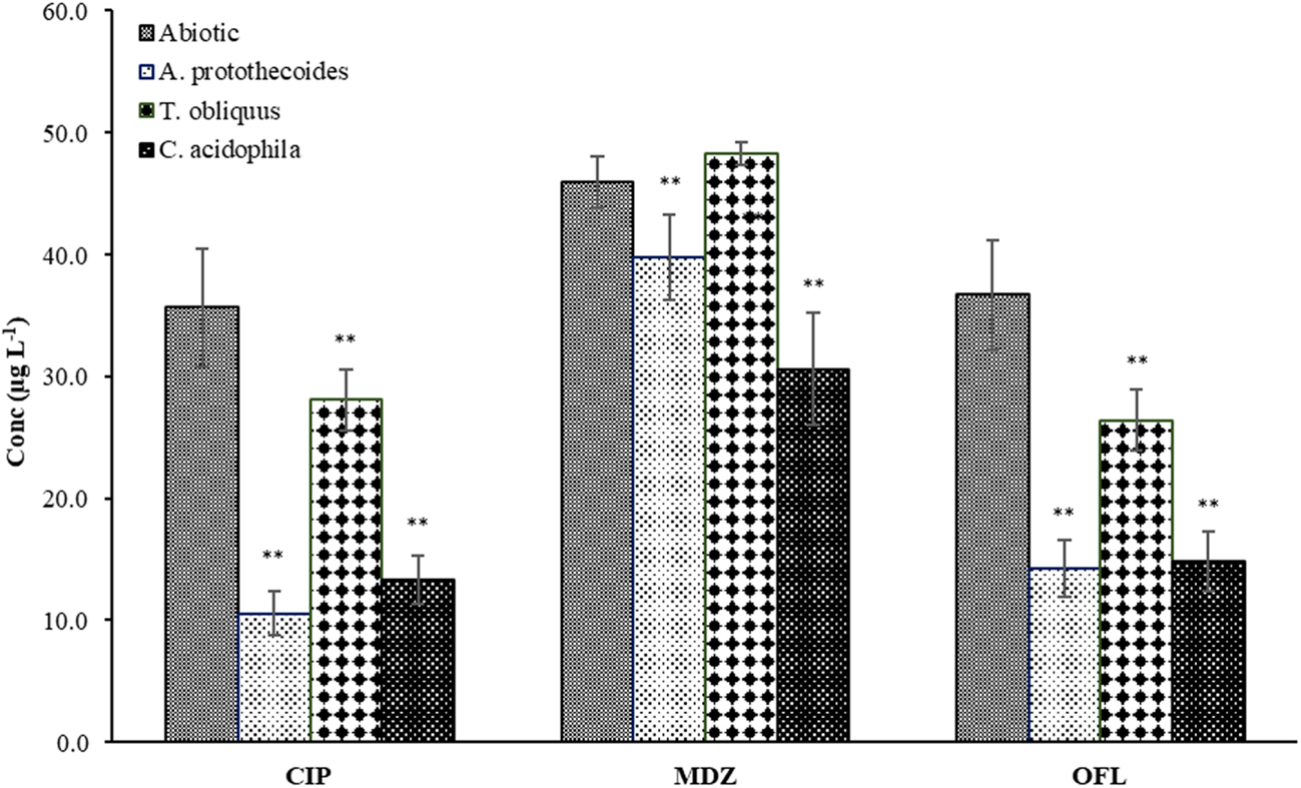
Concentrations of CIP, MDZ, and OFL (μg L^−1^) on day 0 in the abiotic (without microalgae) and biotic (with the three microalgae) experiments. The initial concentration spiked for each antibiotic was 50 μg L^−1^. The figure shows the average data of three samples and three measurements. ** means a statistically significant difference between the abiotic and biotic experiments at 99% (*p* < 0.01)

In this study, antibiotics such as CIP, MDZ, and OFL in Mix 50 appeared to be significantly bioadsorbed by microalgae since their concentrations were immediately lower in biotic samples compared to the abiotic samples after 10 min of contact at the beginning of the experiment (Fig. 4). During the incubation period, the concentrations of the three antibiotics remained constant (Supplementary information, Table S6). This pattern was similar for the three studied microalgae, although the removal efficiencies differed among them. The initial removal rates of CIP ranged between 21 and 70% among the three microalgae, with *A. protothecoides* being the most efficient (Table 3). On the other hand, *C. acidophila* showed more significant adsorption of MDZ (33%), where the efficiencies of the other two microalgae were lower, 13% for *A. protothecoides* and negligible for *T. obliquus*. Regarding OFL, *A. protothecoides* proved to be the most efficient in its removal, with 61% adsorbed from the media. To the best of the authors’ knowledge, this is the first study investigating the ability of *A. protothecoides* to remove antibiotics.

**Table 3:** Biosorption (in % and μg L^−1^) ± SD right at the beginning of the experiment. The values were calculated considering those in the abiotic control as initial concentrations. The table shows the average data of three samples and three measurements ± SD

	*A. protothecoides*	*T. obliquus*	*C. acidophila*
CIP			
Biosorption ± SD (%)	70.4 ± 20.2	21.2 ± 16.2	62.6 ± 21.9
Biosorption ± SD (μg L^−1^)	25.07 ± 5.1	7.54 ± 5.4	22.29 ± 5.2
MDZ			
Biosorption ± SD (%)	13.5 ± 15.7	-4.9 ± 5.0	33.5 ± 10.0
Biosorption ± SD (μg L^−1^)	6.21 ± 4.1	-2.26 ± 2.3	15.37 ± 5.1
OFL			
Biosorption ± SD (%)	61.1 ± 20.4	28.0 ± 15.4	59.5 ± 20.5
Biosorption ± SD (μg L^−1^)	22.42 ± 5.0	10.28 ± 5.1	21.80 ± 5.1

The results obtained in this set of experiments seem to indicate that antibiotics passively bound onto algal cells starting from the first contact with the microorganisms. This observation is consistent with the results of previous studies (Ali et al. 2018; Angulo et al. 2018). In particular, Zambrano et al. (2021), using exhausted biomass composed mainly of algae (*Scenedesmus almeriensis*), demonstrated that biosorption has a significant role in treating antibiotics-rich WW, CIP, and SMX among others, reporting removal percentages varied between 12 and 80%. In another study, the overproduction of exopolymeric substances in *C. vulgaris*, stimulated by the presence of MDZ in the media, was related to the effective adsorption of this antibiotic by the live algal biomass (Hena et al. 2020).

**Fig. 5:**
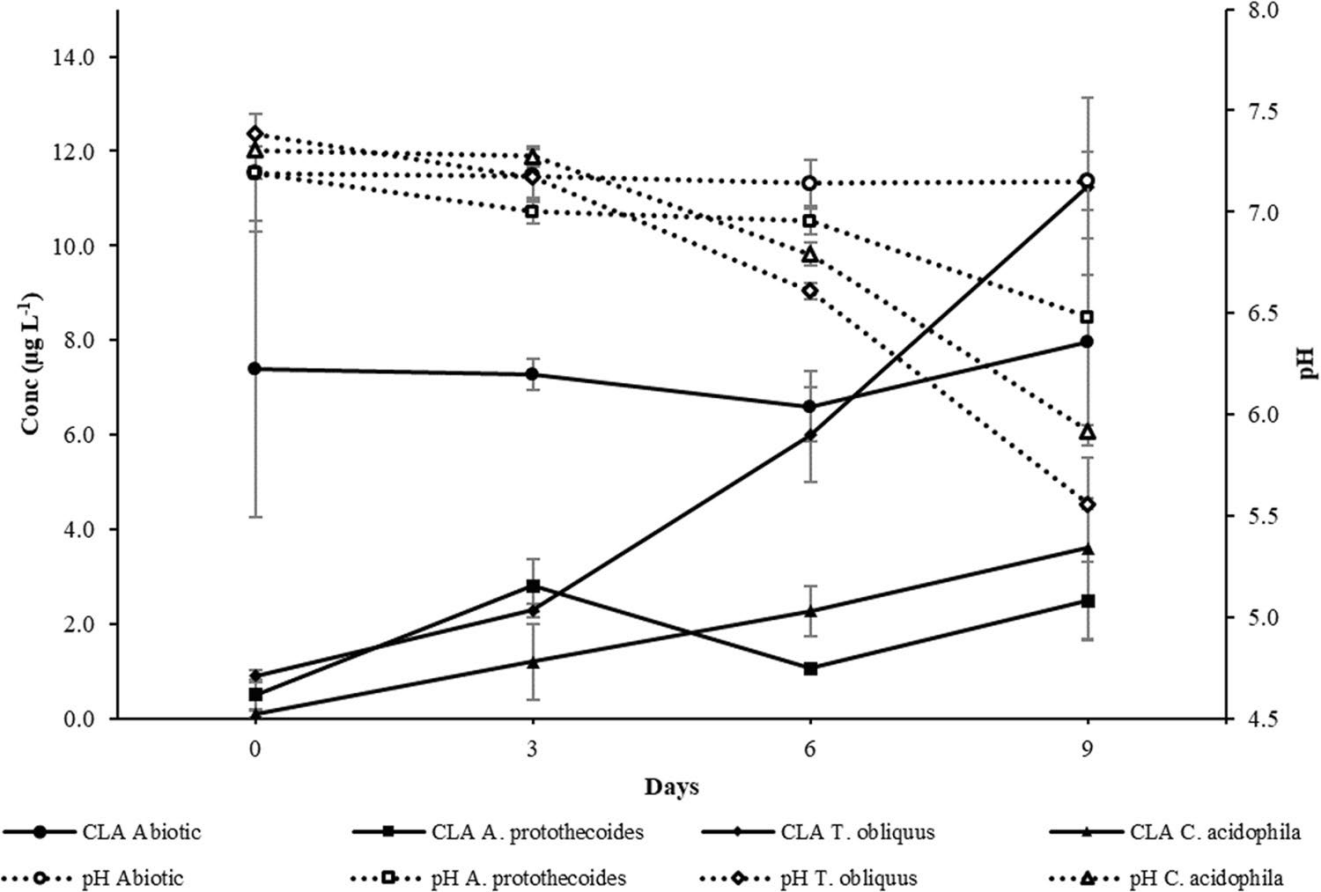
pH variation against the CLA pattern over time for the three species. The initial concentration spiked for each antibiotic was 50 μg L^−1^. It must be noted that a similar pattern can be observed in Mix 100 at a higher degree (Fig. S2 in Supplementary information). The figure shows the average data of three samples and at least one measurement

By contrast, CLA appeared to behave differently in the presence of microalgae during the incubation period (Fig. 5). In this case, although the antibiotic concentration at day 0 was drastically lower than in the abiotic control, it increased progressively during incubation in the media with microalgae. This trend was likely linked to the decrease in pH previously observed (Fig. 1). At D0, the concentration of CLA in the media with the microalgae ranged between 0.1 and 0.9 μg L^−1^, 89 to 99% lower than the abiotic control. At the end of the experiment, it reached values ranging from 2.5 to 11.2 μg L^−1^. The pattern observed could indicate a consistent desorption of the antibiotic due to a pH decrease in the media following the initial biosorption in the algal biomass. The desorption rate was different between the different microalgae studied. After 9 days of incubation, *T. obliquus* released all the CLA adsorbed during day 0, with a final concentration comparable to the abiotic control. On the other hand, by the end of the incubation period, *A. protothecoides* and *C. acidophila* retained a considerable amount of CLA, 69 and 55%, respectively. Other authors have previously reported similar removal rates of CLA in the presence of microalgae. Escudero et al. (2020) demonstrated CLA removal rates of 50–64% using *C. acidophila* in artificial WW. Similarly, in a previous study using four microalgal strains in pre-sterilised synthetic WW, CLA underwent 76% removal on average over 40 days of incubation (Kiki et al. 2020).

The relationship between CLA concentration in the media and the decrease in pH suggests that this pH drop might be the cause of CLA desorption from the biomass into the media, probably due to the changing of the protonation state of the molecule. Vassalle et al. (2020) previously described how the WW pH might contribute to the removal of pharmaceuticals by changing the protonation of their functional groups. The pKa of CLA is 8.99, which indicates that this compound exists in the cation form at pH values of 5 to 9 (McFarland et al. 1997). Since cations generally are adsorbed more strongly to organic carbon sources (Mackay and Boethling 2000), such as the negatively charged surfaces of the algal cells (Pelczar and Reid 1958), the drop in the pH resulted in a reduced capability of the algal biomass to retain the antibiotic. This hypothesis seems to be endorsed by the observations made recently by other colleagues when suggesting that electrostatic repulsion between the charges of the antibiotics and microalgae cell walls plays an essential role in biosorption (García-Galán et al. 2020).

The current study shows that microalgae passively interact with antibiotics in water, instantly removing them from the media via biosorption. In general, *C. acidophila* and *A. protothecoides* showed the highest biosorption potential for most antibiotics commonly found in WW used in this study. It must be noted that biosorption is equally dependent on the two parts involved: algae and antibiotics, and may vary according to the physiological responses of the former to the exogenous compounds in the media. Moreover, this process occurs physically, without the consumption of energy, through the formation of chemical bonding between the functional groups of the cell wall and the antibiotics. Therefore, additional investigation is required to identify these groups and the specific nature of bonds and unravel how this bonding may vary according to the algal physiological responses to antibiotics.

#### Bioaccumulation and biodegradation

If biosorption is a passive mechanism that, as could be observed in the current study, takes place in the first phases of the interaction of the microalgae with antibiotics, bioaccumulation and biodegradation are two active energy-driven processes, which take over later. Bioaccumulation and biodegradation can be distinguished from one another by observing the increase in the degradation products of the antibiotics as a clear manifestation of the latter mechanism. In this study, the production of transformation products has not been investigated. Therefore, a decrease in the antibiotic concentration after day 0—where the biosorption occurs—will be indicated with the general term active removal.

**Fig. 6:**
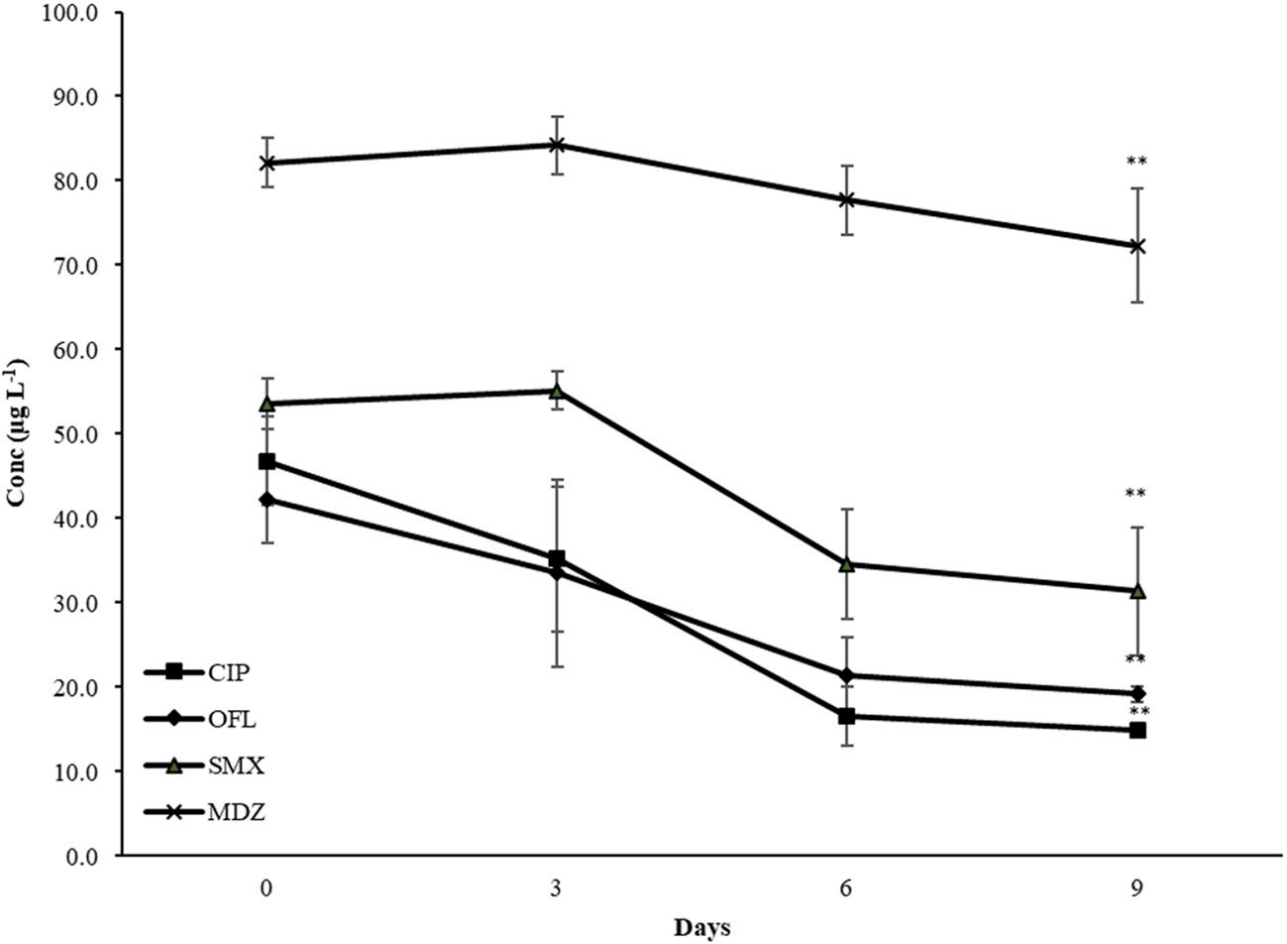
Pattern of CIP, MDZ, OFL, and SMX in the only media in which accumulation was detected (*A. protothecoides*, Mix 100 ). The initial concentration spiked for each antibiotic was 100 μg L^−1^. The figure shows the average data of three samples and three measurements. ** means a statistically significant difference between days 9 and 0 at 99% (*p* < 0.01)

In the current study, an active removal was observed for 4 antibiotics (CIP, MDZ, OFL, and SMX) only in the presence of *A. protothecoides* at the highest concentration of antibiotics (Mix 100) (Fig. 6). In contrast, neither *C. acidophila* nor *T. obliquus* demonstrated active removal, as indicated in Table S7 of the Supplementary information. After 9 days of incubation, the removal efficiencies for *A. protothecoides* were 12, 42, 55, and 69% for MDZ, SMX, OFL, and CIP, respectively (Table 4). As far as the authors know, this is the first time the active removal of antibiotics has been investigated in this species.

**Table 4:** Bioaccumulation and biodegradation indicated as active removal (in % and μg L^−1^) ± SD after 9 days of incubation in Mix 100 with *A. protothecoides*. Values are presented as the difference between day 0 and day 9

	CIP	OFL	SMX	MDZ
Active removal %	68.5 ± 11.4	54.8 ± 13.1	41.6 ± 24.9	12.0 ± 10.0
Active removal μg L^−1^	32.0 ± 5.2	23.2 ± 5.2	22.3 ± 8.2	9.8 ± 7.4

Other species have previously been shown to be efficient in removing these antibiotics over time. When *Chlamydomonas reinhardtii*, *T. obliquus*, *Chlorella pyrenoidosa*, *C. vulgaris*, inoculated in sterilised WW, were exposed to a mixture of 19 antibiotics, including SMX and OFL, the removal percentages ranged between 43 and 52% for OFL and did not exceed 78% for CIP (Zhou et al. 2014). On the other hand, Kiki et al. (2020), using a similar experimental setup and testing another four strains of freshwater microalgae, reported removal percentages for SMX ranging from 21 to 97% after 40 days.

The reported findings show that only *A. protothecoides* actively remove antibiotics in the media with the highest concentration (Mix 100). It appears that *A. protothecoides* might start actively accumulating or degrading the antibiotics only when they reach a specific concentration able to boost the heterotrophic mode, as suggested by the change in the pH of the media, particularly accentuated in Mix 100. *A. protothecoides*, compared to the other two species employed in this study, is broadly recognised to perform better in heterotrophic and mixotrophic conditions where the presence of organic substances may enhance growth and productivity (Xu et al. 2006; Heredia-Arroyo et al. 2010; Joun et al. 2021). According to the results of this study, it seems that this species may actively interact with antibiotics, according to their concentrations, by raising the heterotrophic activity and degrading them for nutritional purposes. This may also explain the high overproduction of pigments—especially ChlB—of *A. protothecoides* (Fig. 3, b), hypothetically produced to reduce induced stress (Sun et al. 2018) or degrade the exogenous compounds (Luo et al. 2015).

The detection of the transformation products to distinguish between the two active processes of bioaccumulation and biodegradation was not investigated in this study. Therefore, further research is needed to discern better the relative weight of the two parts in active removal in the presence of microalgae.

## Conclusions

The 3 studied microalgae species (*A. protothecoides, T. obliquus, and C. acidophila*) efficiently removed antibiotics from the media, suggesting microalgae-based technology as a solution for antibiotics-containing WW. These algae showed no growth inhibition, even under high-stress conditions.

*A. protothecoides* and *C. acidophila* were particularly effective in adsorbing antibiotics like CIP, CLA, MDZ, and OFL. Moreover, *A. protothecoides* actively removed SMX, CIP, MDZ, and OFL over time, likely due to increased heterotrophic activity triggered by antibiotic concentrations. This previously unstudied species displayed promising potential for treating antibiotics-containing WW.

Stress responses in microalgae were observed, with *A. protothecoides* exhibiting overproduction of pigments, especially ChlB, in response to antibiotic exposure. The pH decrease in the media was another response to the stress, and it regulated the removal of certain antibiotics, such as CLA.

This study represents an initial exploration, providing insight into the interactive effects of physiological responses to antibiotics and their removal in microalgae. Further long-term continuously fed experiments are needed to better assess the impact of these antibiotics on microalgae performance and potential inhibition.

### Supplementary information

ESM 1 (DOCX 230 kb)

## Data Availability

The authors declare that the data supporting the findings of this study are available within the paper and its Supplementary Information files. Should any raw data files be needed in another format they are available from the corresponding author upon reasonable request.
