# Supplementary material for: Removal efficiencies of seven frequently detected antibiotics and related physiological responses in three microalgae species
Source: Environ Sci Pollut Res Int. 2024 Jan 26;31(9):14178–90. doi: 10.1007/s11356-024-32026-5 (PMC10881744; doi:10.1007/s11356-024-32026-5)
Supplement: Supplementary file 1 — (DOCX 230 kb) [file 11356_2024_32026_MOESM1_ESM.docx]

**Supplementary Information**

Removal efficiencies of seven frequently detected antibiotics and related physiological responses in three microalgae species

Gabriele Frascaroli ^1*^, Joanne Roberts ^2^, Colin Hunter ^1^ and Ania Escudero ^1^

^1^ Department of Civil Engineering and Environmental Management, School of Computing, Engineering and Built Environment, Glasgow Caledonian University, Cowcaddens Road, Glasgow G4 0BA, Scotland, UK

^2^ Department of Applied Science, School of Computing, Engineering and Built Environment, Glasgow Caledonian University, Cowcaddens Road, Glasgow G4 0BA, Scotland, UK

^*^ Author to whom correspondence should be addressed at [gabriele.frascaroli@gcu.ac.uk](mailto:gabriele.frascaroli@gcu.ac.uk).

**Table S1**. Bg-11 Medium composition with concentrations of principal salts as to WW.

| **Compound** | **Concentration mg L^-1^** |
| --- | --- |
| (NH_4_)_2_SO_4_ | 241.7 |
| NaNO_3_ | 1.8 |
| K_2_HPO_4_ | 28.1 |
| MgSO_4_.7H_2_O | 75 |
| CaCl_2_.2H_2_O | 36 |
| Na_2_CO_3_ | 20 |
| Citric acid | 6 |
| Ammonium iron(III) citrate | 6 |
| Na_2_EDTA | 1 |
| **Trace metal solution**  H_3_BO_3_  ZnCl_2_  MnCl_2_.4H_2_O  Na_2_MoO_4_·2H_2_O  CuSO_4_.5H_2_O  Co(NO_3_)_2_·6H_2_O | 2.86  4.64  0.39  0.22  0.08  0.05 |

**Figure S1**. SPE method Oasis HLB 3 cc cartridges (60 mg sorbent, 30 µm). Reconstitution was made with 0.5, 1 or 1.5 mL CH_3_CN/18.2 mΩ H2O 10/90 for samples with spiked concentrations of 10, 50 and 100 µg L^-1^, respectively.

**Table S2.** Recoveries for the seven antibiotics. Recoveries were calculated following the SPE method as described before. Mixtures of antibiotics (50 µg L^-1^) were spiked to modified BG-11 media, and samples were passed through SPE. The resulting concentrations were compared to a blank matrix composed of Acet/18.2 mΩ H2O 10/90 spiked with the same antibiotic mixture at the identical concentration.

| Antibiotic | Recovery |
| --- | --- |
| Ciprofloxacin | 79.30% |
| Clarithromycin | 80.52% |
| Erythromycin | 12.53% |
| Metronidazole | 102.05% |
| Ofloxacin | 68.87% |
| Sulfamethoxazole | 70.32% |
| Trimethoprim | 96.98% |

**Table S3**. Matrix effect for the seven antibiotics. Matrix effect was calculated following the SPE method as described before. Mixtures of antibiotics (50 µg L^-1^) were spiked to modified BG-11 media and compared to a blank matrix of water spiked at the same concentrations.

| Antibiotic | Average Amt (µg L^-1^)  Solvent | Average Amt (µg L^-1^)  Matrix | Matrix Effect  (%)* |
| --- | --- | --- | --- |
| Ciprofloxacin | 41.3 | 41.7 | 101.00 |
| Clarithromycin | 49.5 | 36.5 | 73.65 |
| Erythromycin | 48.6 | 43.4 | 89.35 |
| Metronidazole | 49.5 | 46.6 | 94.07 |
| Ofloxacin | 45.3 | 48.7 | 107.48 |
| Sulfamethoxazole | 48.2 | 45.8 | 94.96 |
| Trimethoprim | 46.7 | 42.7 | 91.41 |

* Matrix effect expressed as the ratio of the average amount of an analyte spiked in the solvent to the the ratio of the average amount of an analyte spiked in the matrix multiplied by 100.

**Table S4**. Limit of Quantification (LOQ). All analytes' calibration curves were linear with a 1/X^2^ weighting, and calibration points with deviation greater than +/- 20% from the best fit were removed. Using this criterion, the LOQ was set at the lowest concentration of the standard curve that could be measured within these limits and the upper limit of quantitation (ULOQ) at the highest. A minimum of 4 points was required for a calibration line. The data was not forced through zero; the calibration lines and quantitation were carried out using the instrument software.

| Antibiotic | LOQ (µg L^-1^) | ULOQ (µg L^-1^) |
| --- | --- | --- |
| Ciprofloxacin | 0.25 | 25 |
| Clarithromycin | 0.10 | 100 |
| Erythromycin | 0.25 | 100 |
| Metronidazole | 0.10 | 100 |
| Ofloxacin | 0.25 | 25 |
| Sulfamethoxazole | 0.10 | 100 |
| Trimethoprim | 0.25 | 50 |

LC-MS Conditions (S5)

The antibiotic concentrations in the media were analysed using liquid chromatography coupled with mass spectrometry (LC-MS) using a Thermo Scientific Q-Exactive Orbitrap mass spectrometer equipped with an Accucore™ C18 HPLC Column (150 × 2.1 mm) (Thermo Fisher Scientific, USA). The mass spectrometer was fitted with a Dionex Ultimate 3000 RS pump, Dionex Ultimate 3000 RS autosampler (temperature controlled at 10 °C) and Dionex Ultimate 3000 RS column compartment (temperature controlled at 30 °C). The operating software was Chromeleon, Xcalibur and Tracefinder. The antibiotics were detected in positive mode. The mobile phase A for the chromatographic separation was acetonitrile, and the mobile phase B was 10 mM ammonium formate in water 18 mΩ (adjusted to pH 3.5 with formic acid). The gradient elution was performed at a constant flow rate as follows: mobile phase B was retained at 99 %, decreased to 65 % B over 2 min, maintained at 65 % B for 5 min, then decreased to 1 % B over 4 min. The gradient was maintained at 1 % B for a further 6 min, then increased back to 99 % B over 1 min and equilibrated at 99% B for 9 min. The flow was 0.2 mL/min, and the injection volume was 10 μL.

**Table S5**. Molecular formula, exact mass, precursor, mass accuracy calculation and retention time
for the 7 antibiotics.

| **Antibiotic** | **Molecular formula** | **Exact mass**  [M+H]^+^ | **Precursor**  [M+H]^+^ | **Product Ion** | **Retention Time** |
| --- | --- | --- | --- | --- | --- |
| Ciprofloxacin | C_17_H_18_FN_3_O_3_ | 331.3415 | 332.1390 | 288.1499 | 6.71 |
| Clarithromycin | C_38_H_69_NO_13_ | 747.9534 | 748.4822 | 158.1172 | 9.82 |
| Ofloxacin | C_18_H_20_FN_3_O_4_ | 361.3730 | 362.1511 | 318.1616 | 6.69 |
| Metronidazole | C_6_H_9_N_3_O_3_ | 171.1540 | 172.0721 | 128.0460 | 6.04 |
| Trimethoprim | C_14_H_18_N_4_O_3_ | 290.3180 | 291.1455 | 245.1043 | 6.65 |
| Erythromycin | C_37_H_67_NO_13_ | 733.9268 | 734.4660 | 158.1172 | 8.06 |
| Sulfamethoxazole | C_10_H_11_N_3_O_3_S | 253.2790 | 254.0593 | 156.0111 | 7.73 |

**Figure S2**. Growth curves of the three microalgae when exposed to three antibiotic mixtures at 10, 50, and 100 µg L^-1^, compared to the control. A. protothecoides and T. obliquus showed no inhibition, while C. acidophila exhibited a significant increase in growth in media with higher antibiotic concentrations, possibly due to the increased acidity of the media

**
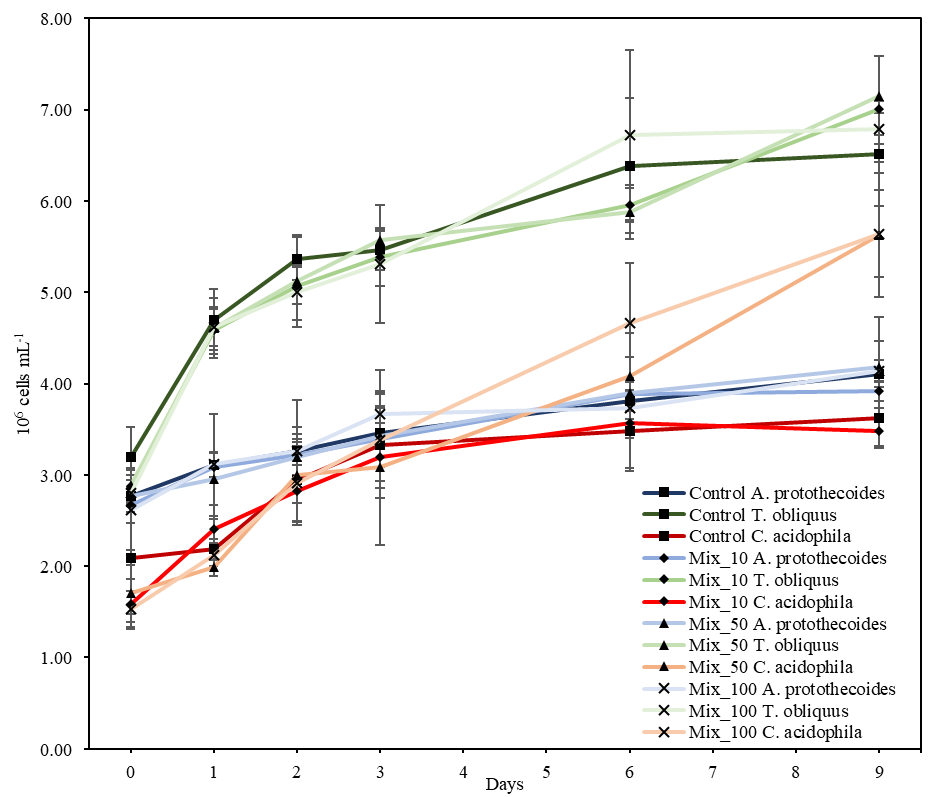
**

**Table S6.** Concentrations (µg L^-1^) for the seven antibiotics in abiotic batches (Dark, Light) and Biotic (with the three algae) considering the dilution factor applied during the reconstitution (Table S2). The initial concentration spiked for each antibiotic was 50 µg L^-1^.

| **Ciprofloxacin Average Conc (µg L^-1^)** | | | | | | **Ciprofloxacin Standard Deviation (± µg L^-1^)** | | | | |
| --- | --- | --- | --- | --- | --- | --- | --- | --- | --- | --- |
|  | **Dark** | **Light** | ***T. obliq*** | ***C. acidop*** | ***A. proto*** | **Dark** | **Light** | ***T. obliq*** | ***C. acidop*** | ***A. proto*** |
| **Day 0** | 36.4 | 34.8 | 28.1 | 13.3 | 10.5 | 1.6 | 8.0 | 2.5 | 2.0 | 1.8 |
| **Day 3** | 51.9 | 46.7 | 21.5 | 15.2 | 12.6 | 3.0 | 2.4 | 6.6 | 1.3 | 7.6 |
| **Day 6** | 37.4 | 36.1 | 32.9 | 12.7 | 8.3 | 2.1 | 3.1 | 2.4 | 1.3 | 0.9 |
| **Day 9** | 33.3 | 31.1 | 23.0 | 10.5 | 8.5 | 1.4 | 10.7 | 1.3 | 6.9 | 2.7 |
| **Clarithromycin Average Conc (µg L^-1^)** | | | | | | **Clarithromycin Standard Deviation (± µg L^-1^)** | | | | |
|  | **Dark** | **Light** | ***T. obliq*** | ***C. acidop*** | ***A. proto*** | **Dark** | **Light** | ***T. obliq*** | ***C. acidop*** | ***A. proto*** |
| **Day 0** | 7.5 | 7.3 | 0.9 | 0.1 | 0.5 | 3.5 | 2.8 | 0.1 | 0.1 | 0.3 |
| **Day 3** | 8.4 | 6.2 | 2.3 | 1.2 | 2.8 | 0.4 | 0.3 | 0.1 | 0.8 | 0.6 |
| **Day 6** | 5.8 | 7.4 | 6.0 | 2.3 | 1.1 | 0.9 | 0.5 | 1.0 | 0.5 | 0.1 |
| **Day 9** | 7.0 | 8.9 | 11.2 | 3.6 | 2.5 | 2.6 | 1.7 | 1.9 | 1.9 | 0.8 |
| **Erythromycin Average Conc (µg L^-1^)** | | | | | | **Erythromycin Standard Deviation (± µg L^-1^)** | | | | |
|  | **Dark** | **Light** | ***T. obliq*** | ***C. acidop*** | ***A. proto*** | **Dark** | **Light** | ***T. obliq*** | ***C. acidop*** | ***A. proto*** |
| **Day 0** | <LOQ * | 0.8 | 0.9 | <LOQ * | <LOQ * | N/A | 2.0 | 0.0 | N/A | 0.1 |
| **Day 3** | <LOQ * | <LOQ * | 0.2 | <LOQ * | <LOQ * | N/A | N/A | 0.0 | N/A | 0.0 |
| **Day 6** | 0.3 | 0.3 | 0.3 | <LOQ * | <LOQ * | 0.1 | 0.1 | 0.0 | N/A | N/A |
| **Day 9** | 0.9 | 0.2 | 0.7 | <LOQ * | <LOQ * | 0.4 | 0.7 | 0.1 | N/A | 0.0 |
| **Metronidazole Average Conc (µg L^-1^)** | | | | | | **Metronidazole Standard Deviation (± µg L^-1^)** | | | | |
|  | **Dark** | **Light** | ***T. obliq*** | ***C. acidop*** | ***A. proto*** | **Dark** | **Light** | ***T. obliq*** | ***C. acidop*** | ***A. proto*** |
| **Day 0** | 47.9 | 44.0 | 48.2 | 30.6 | 39.7 | 1.8 | 2.4 | 0.9 | 4.6 | 3.5 |
| **Day 3** | 47.6 | 43.6 | 49.8 | 35.9 | 43.9 | 0.3 | 1.0 | 2.1 | 3.2 | 3.4 |
| **Day 6** | 46.3 | 44.6 | 48.6 | 36.4 | 36.2 | 1.0 | 1.9 | 0.9 | 1.2 | 1.1 |
| **Day 9** | 49.9 | 47.8 | 49.9 | 31.4 | 39.5 | 0.8 | 0.7 | 1.2 | 4.1 | 1.4 |
| **Ofloxacin Average Conc (µg L^-1^)** | | | | | | **Ofloxacin Standard Deviation (± µg L^-1^)** | | | | |
|  | **Dark** | **Light** | ***T. obliq*** | ***C. acidop*** | ***A. proto*** | **Dark** | **Light** | ***T. obliq*** | ***C. acidop*** | ***A. proto*** |
| **Day 0** | 36.5 | 36.8 | 26.4 | 14.9 | 14.3 | 5.5 | 3.4 | 2.5 | 2.4 | 2.4 |
| **Day 3** | 47.3 | 42.5 | 19.5 | 16.6 | 12.6 | 3.3 | 3.1 | 9.8 | 0.8 | 7.3 |
| **Day 6** | 43.7 | 37.5 | 34.2 | 15.4 | 12.1 | 11.0 | 4.6 | 3.9 | 1.8 | 1.1 |
| **Day 9** | 34.2 | 29.7 | 23.8 | 12.0 | 10.6 | 2.4 | 7.5 | 2.8 | 7.0 | 1.4 |
| **Sulfamethoxazole Average Conc (µg L^-1^)** | | | | | | **Sulfamethoxazole Standard Deviation (± µg L^-1^)** | | | | |
|  | **Dark** | **Light** | ***T. obliq*** | ***C. acidop*** | ***A. proto*** | **Dark** | **Light** | ***T. obliq*** | ***C. acidop*** | ***A. proto*** |
| **Day 0** | 30.1 | 37.6 | 25.5 | 23.0 | 19.0 | 2.9 | 9.8 | 5.5 | 4.1 | 2.1 |
| **Day 3** | 33.4 | 32.9 | 26.2 | 28.2 | 24.6 | 2.4 | 2.6 | 3.8 | 4.9 | 4.0 |
| **Day 6** | 31.4 | 30.4 | 30.0 | 29.3 | 12.9 | 1.2 | 2.7 | 2.1 | 6.6 | 0.6 |
| **Day 9** | 35.8 | 28.2 | 30.0 | 26.2 | 16.8 | 4.3 | 9.3 | 0.8 | 10.2 | 1.8 |
| **Trimethoprim Average Conc (µg L^-1^)** | | | | | | **Trimethoprim Standard Deviation (± µg L^-1^)** | | | | |
|  | **Dark** | **Light** | ***T. obliq*** | ***C. acidop*** | ***A. proto*** | **Dark** | **Light** | ***T. obliq*** | ***C. acidop*** | ***A. proto*** |
| **Day 0** | 36.2 | 33.0 | 33.4 | 27.4 | 30.2 | 2.3 | 3.4 | 1.5 | 4.8 | 2.7 |
| **Day 3** | 62.7 | 38.2 | 35.0 | 32.0 | 30.5 | 37.3 | 7.5 | 1.7 | 1.7 | 2.7 |
| **Day 6** | 53.6 | 43.0 | 33.1 | 32.5 | 30.2 | 22.3 | 14.4 | 2.5 | 2.5 | 3.0 |
| **Day 9** | 40.4 | 38.3 | 35.6 | 28.0 | 31.8 | 5.3 | 3.2 | 3.0 | 4.7 | 2.7 |

* LOQ: 0.25 µg L^-1^.

N/A: Not Applicable

**Figure S3**. Changes in CLA concentration in relation to the pH values in samples spiked with 100 µg L^-1^ of each antibiotic.


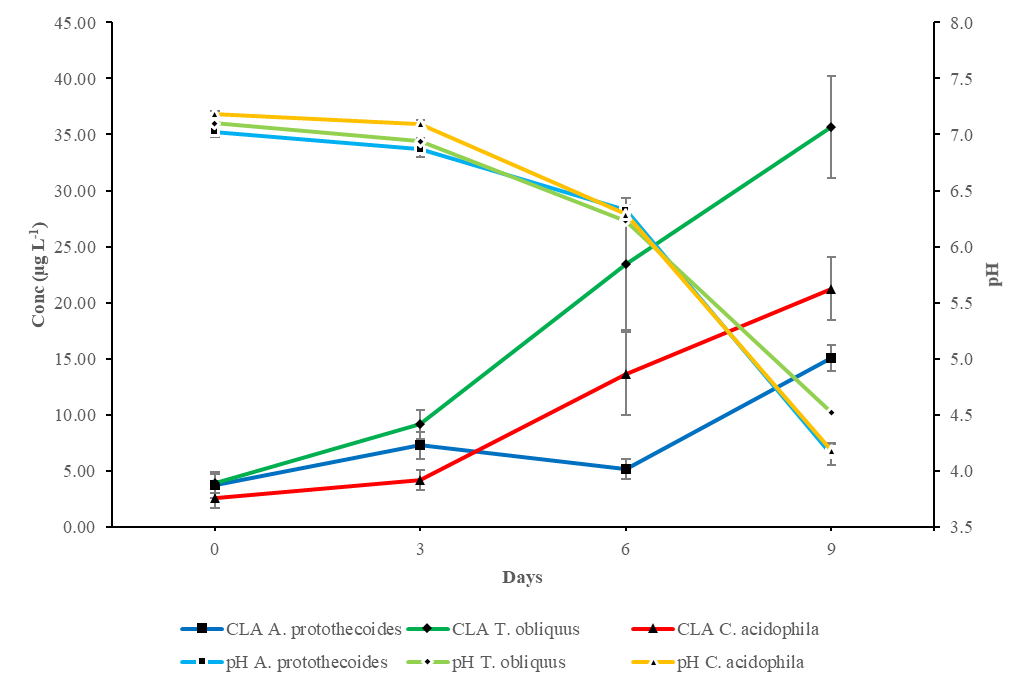


**Table S7.** Concentrations (µg L^-1^) for the seven antibiotics in biotic batches considering the dilution factor applied during the reconstitution (Table S2). The initial concentration spiked for each antibiotic was 100 µg L^-1^.

| **Ciprofloxacin Average Conc (µg L^-1^)** | | | | **Ciprofloxacin Standard Deviation (± µg L^-1^)** | | |
| --- | --- | --- | --- | --- | --- | --- |
|  | ***T. obliq*** | ***C. acidop*** | ***A. proto*** | ***T. obliq*** | ***C. acidop*** | ***A. proto*** |
| **Day 0** | 62.12 | 32.08 | 46.74 | 2.91 | 4.41 | 5.21 |
| **Day 3** | 57.54 | 37.54 | 35.12 | 8.01 | 12.33 | 8.57 |
| **Day 6** | 62.40 | 24.14 | 16.55 | 16.66 | 16.47 | 3.51 |
| **Day 9** | 68.68 | 26.16 | 14.74 | 7.56 | 7.18 | 0.32 |
| **Clarithromycin Average Conc (µg L^-1^)** | | | | **Clarithromycin Standard Deviation (± µg L^-1^)** | | |
|  | ***T. obliq*** | ***C. acidop*** | ***A. proto*** | ***T. obliq*** | ***C. acidop*** | ***A. proto*** |
| **Day 0** | 3.85 | 2.54 | 3.74 | 0.85 | 0.85 | 1.18 |
| **Day 3** | 9.13 | 4.13 | 7.26 | 1.30 | 0.88 | 1.22 |
| **Day 6** | 23.44 | 13.66 | 5.15 | 5.86 | 3.69 | 0.90 |
| **Day 9** | 35.64 | 21.25 | 15.06 | 4.56 | 2.80 | 1.16 |
| **Erythromycin Average Conc (µg L^-1^)** | | | | **Erythromycin Standard Deviation (± µg L^-1^)** | | |
|  | ***T. obliq*** | ***C. acidop*** | ***A. proto*** | ***T. obliq*** | ***C. acidop*** | ***A. proto*** |
| **Day 0** | 1.45 | <LOQ * | 0.02 | 0.05 | N/A | 0.04 |
| **Day 3** | 0.56 | <LOQ * | 0.12 | 0.12 | N/A | 0.05 |
| **Day 6** | 1.19 | <LOQ * | 0.23 | 0.31 | N/A | 0.07 |
| **Day 9** | 1.77 | <LOQ * | 0.61 | 0.23 | N/A | 0.09 |
| **Metronidazole Average Conc (µg L^-1^)** | | | | **Metronidazole Standard Deviation (± µg L^-1^)** | | |
|  | ***T. obliq*** | ***C. acidop*** | ***A. proto*** | ***T. obliq*** | ***C. acidop*** | ***A. proto*** |
| **Day 0** | 93.26 | 62.54 | 82.10 | 7.61 | 7.14 | 2.91 |
| **Day 3** | 102.50 | 73.06 | 84.18 | 5.57 | 6.50 | 3.45 |
| **Day 6** | 97.60 | 72.04 | 77.70 | 3.21 | 9.90 | 4.10 |
| **Day 9** | 100.83 | 64.97 | 72.28 | 4.01 | 8.96 | 6.77 |
| **Ofloxacin Average Conc (µg L^-1^)** | | | | **Ofloxacin Standard Deviation (± µg L^-1^)** | | |
|  | ***T. obliq*** | ***C. acidop*** | ***A. proto*** | ***T. obliq*** | ***C. acidop*** | ***A. proto*** |
| **Day 0** | 57.34 | 34.95 | 42.24 | 3.79 | 3.51 | 5.16 |
| **Day 3** | 59.96 | 40.52 | 33.44 | 9.07 | 5.42 | 11.09 |
| **Day 6** | 65.40 | 25.14 | 21.39 | 15.93 | 16.28 | 4.38 |
| **Day 9** | 65.22 | 27.79 | 19.09 | 10.00 | 7.98 | 0.89 |
| **Sulfamethoxazole Average Conc (µg L^-1^)** | | | | **Sulfamethoxazole Standard Deviation (± µg L^-1^)** | | |
|  | ***T. obliq*** | ***C. acidop*** | ***A. proto*** | ***T. obliq*** | ***C. acidop*** | ***A. proto*** |
| **Day 0** | 47.05 | 52.66 | 53.56 | 7.15 | 9.35 | 3.01 |
| **Day 3** | 53.78 | 63.65 | 55.07 | 7.70 | 11.46 | 2.24 |
| **Day 6** | 64.20 | 62.82 | 34.51 | 1.75 | 13.78 | 6.53 |
| **Day 9** | 54.81 | 44.16 | 31.27 | 4.89 | 6.57 | 7.58 |
| **Trimethoprim Average Conc (µg L^-1^)** | | | | **Trimethoprim Standard Deviation (± µg L^-1^)** | | |
|  | ***T. obliq*** | ***C. acidop*** | ***A. proto*** | ***T. obliq*** | ***C. acidop*** | ***A. proto*** |
| **Day 0** | 64.00 | 55.53 | 59.67 | 8.40 | 4.02 | 5.43 |
| **Day 3** | 66.61 | 64.71 | 59.80 | 5.06 | 6.53 | 5.01 |
| **Day 6** | 62.92 | 62.54 | 59.90 | 5.37 | 9.25 | 4.34 |
| **Day 9** | 63.81 | 60.53 | 55.98 | 4.05 | 11.00 | 7.39 |

* LOQ: 0.25 µg L^-1^

N/A: Not Applicable
